# Relationships Between Genetic Parameters in the Component Traits of a Ratio Trait and the Distribution and Heritability of Such Ratio Trait

**DOI:** 10.1111/asj.70031

**Published:** 2025-01-22

**Authors:** Masahiro Satoh

**Affiliations:** ^1^ Graduate School of Agricultural Science Tohoku University Sendai Japan

**Keywords:** component traits, genetic parameter, heritability, phenotypic distribution, ratio trait

## Abstract

The relationships between the distribution and heritability of a ratio trait and the genetic parameters of its component traits were clarified using a Monte Carlo computer simulation. The component traits of a ratio trait were assumed to be normally distributed. The genetic parameters of the ratio trait were estimated using parent–offspring regression. As the genetic and environmental correlations ($r_{G}$ and $r_{E}$) of the component traits increased, the variance, skewness, and kurtosis of the ratio trait decreased. The amount of decrease had a greater effect on $r_{E}$ than on $r_{G}$. The estimated heritability of the ratio trait decreased as $r_{G}$ increased and as $r_{E}$ decreased. The estimated heritability of a ratio trait can be higher than the heritability of any of its component traits.

## 1 Introduction

Feed efficiency (FE), the ratio of weight gain to feed intake, is one of the most economically important characters in livestock production. Percentage traits such as fat and protein yield are also a type of ratio trait. Many economic traits in livestock production are expressed as a ratio of two component traits. There are numerous examples of estimating genetic parameters to use these traits for selection (e.g., feed availability: Hoque et al. 2009; Do et al. 2013; milk fat percentage: Butcher, Sargent, and Legates 1967; Henk and Groen 1998). However, if the two component traits in a ratio trait are normally distributed, the ratio trait will not be normally distributed (Hinkley 1969; Marsaglia 2006).

Selection for ratio traits has been discussed for many years (e.g., Turner 1959). The distribution of the phenotypic value of a ratio trait depends on the genetic parameters of its component traits. However, even if the breeding values of the component traits in animals are known, the breeding values of the ratio trait are not. Furthermore, there have been no studies that clarify the relationship between the heritability of the ratio trait and the genetic parameters of its component traits.

The objective of this study is to clarify the relationships between the distribution and heritability of a ratio trait and the genetic parameters of its component traits using a Monte Carlo computer simulation.

## 2 Material and Methods

### 2.1 Distribution of the Ratio Trait

The two component traits of the ratio trait (Trait 0) are Traits 1 and 2. These two traits are assumed to be normally distributed. Thus,

$$\text{Trait}\;0\;\left(\text{ratio trait}\right)=\text{Trait}\;1/\text{Trait}\;2.$$

First, to clarify the relationship between the distribution of Trait 0 and the mean and coefficients of variation of Traits 1 and 2 (CV1 and CV2), 2,000,000 records of the ratio trait from 2,000,000 independent individuals were generated using a Monte Carlo simulation. The simulation conditions were (1) the ratio of the means of Traits 1 and 2 (Trait 1/Trait 2) = 10/25, 10/15, 10/10, 15/10, 20/10, and 25/10 (seven combinations) and (2) all combinations of the coefficients of variation (CV1, CV2) = 0.05, 0.10, and 0.15 for Traits 1 and 2 (nine combinations). For each of the 63 (7×9) combinations, 100 iterations of the simulation were performed to obtain the mean, variance, skewness, and kurtosis of Trait 0.

### 2.2 Distribution and Heritability of a Ratio Trait With Contributing Traits That Have Different Genetic Parameters

The relationships between the genetic parameters of Traits 1 and 2 and the distribution of Trait 0 were examined. A Monte Carlo method was used to simulate two generations of records for each trait. The base generation (G0) of 100,000 males and 1,000,000 females was assumed to be unrelated, unselected, and not inbred. One male was mated with 10 females, and each female in G0 produced two offspring (G1). The population size of G1 was therefore 2,000,000.

The phenotypic value ($p_{\mathrm{ij}}$) of Trait *j* (j=1,2) for the *i* ^th^ individual in G0 is

$$p_{\mathrm{ij}}=\mu_{j}+g_{\mathrm{ij}}+e_{\mathrm{ij}}$$

where $\mu_{j}$ is the mean, $g_{\mathrm{ij}}$ is the breeding value, $e_{\mathrm{ij}}$ is the random error, and Traits 1 and 2 are assumed to be simultaneously normally distributed. Then, $g_{\mathrm{ij}}$ in G1 was generated by

$$g_{i1}=\frac{g_{si1}+g_{di1}}{2}+\sqrt{0.5}\cdot L_{g11}z_{i1},$$

$$g_{i2}=\frac{g_{si2}+g_{di2}}{2}+\sqrt{0.5}\cdot \left(L_{g21}z_{i1}+L_{g22}z_{i2}\right)$$

where $g_{s\mathrm{ij}}$ and $g_{d\mathrm{ij}}$ are the breeding values of Trait *j* for the sire and dam of the *i* ^th^ individual, $L_{g\mathrm{kl}}$ is the element of the *k* ^th^ row and *l* ^th^ column in the lower triangular matrix obtained by Cholesky decomposition of the genetic variance–covariance matrix of Traits 1 and 2, and $z_{i1}$ and $z_{i2}$ are random numbers following an independent standard normal distribution. The $p_{\mathrm{ij}}$ in G1 was generated by

$$p_{i1}=g_{i1}+L_{e11}z_{i3},$$

$$p_{i2}=g_{i2}+\left(L_{e21}z_{i3}+L_{e22}z_{i4}\right)$$

where $L_{e\mathrm{kl}}$ is the element of the *k* ^th^ row and *l* ^th^ column in the lower triangular matrix obtained by Cholesky decomposition of the environment variance–covariance matrix of Traits 1 and 2, and $z_{i3}$ and $z_{i4}$ are random numbers following an independent standard normal distribution. The phenotypic value of Trait 0 for the *i* ^th^ individual was calculated using

$$p_{i0}=\frac{p_{i1}}{p_{i2}}.$$

The phenotypic values of Traits 1 and 2 were both set to a mean of 10 and variance of 1. In addition, 100 iterations of the simulation were performed for all 81 combinations where the heritabilities ($h_{1}^{2}$ and $h_{2}^{2}$) of Traits 1 and 2 were 0.1, 0.3, and 0.5 (9 combinations), respectively, and the genetic correlation ($r_{G}$) and environmental correlation ($r_{E}$) between Traits 1 and 2 were −0.5, 0, and 0.5 (9 combinations), respectively. The mean, variance, skewness, and kurtosis of the phenotypic values of Trait 0 in G1 were calculated. The genetic parameters of Trait 0 were calculated using the following formula to estimate its heritability.

$$\sigma_{g_{0}}^{2}=2\cdot \mathrm{cov}\left(\frac{p_{s0}+p_{d0}}{2},p_{o0}\right),\;\sigma_{p_{0}}^{2}=2\cdot \mathrm{var}\left(\frac{p_{s0}+p_{d0}}{2}\right),$$

where $\sigma_{g_{0}}^{2}$ and $\sigma_{p_{0}}^{2}$ are the additive genetic and phenotypic variances of Trait 0, and $p_{s0}$, $p_{d0}$, and $p_{o0}$ are, respectively, the phenotypic values of the sire, dam, and their offspring.

## 3 Results and Discussion

### 3.1 Distribution of Ratio Trait

**FIGURE 1:**
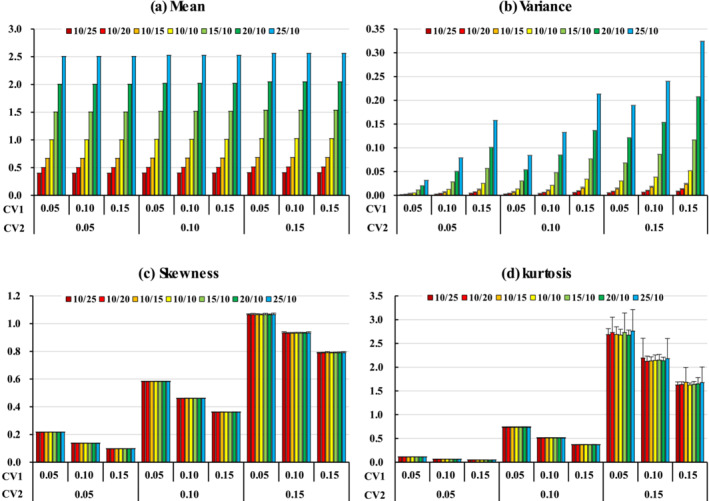
The mean, standard deviation, skewness, and kurtosis as well as the standard deviations of the ratio trait (Trait 0) with the various means and CVs of the component traits (Traits 1 and 2).

Figure 1 shows the mean, standard deviation, skewness, and kurtosis, as well as the standard deviations for the ratio trait (Trait 0) with the various means and CVs of the component traits (Traits 1 and 2). The mean of Trait 0 was very similar to the ratio of the means of Traits 1 and 2 and was not affected by the magnitude of CV1. However, the mean of Trait 0 tended to become slightly higher with increased CV2. Lin (1980) stated that the mean of the ratio trait can approximate the ratio of the expected value of the component traits. The variance of Trait 0 rose with increased ratios of the means of Traits 1 and 2. It also increased as CV1 and CV2 increased. Budylin et al. (2018) reported that the variance of the ratio trait increased as the variance of the component traits increased. The results of this study were consistent with these observations.

Neither the skewness nor the kurtosis of Trait 0 was affected by differences in the ratio of the means of Traits 1 and 2. This is because they were compared with the same CV. Both the skewness and kurtosis of Trait 0 were positive values, and the distribution had a right tail with a sharp center. Furthermore, skewness and kurtosis increased on decreasing CV1 and increasing CV2, with the effect of CV2 being particularly notable. The standard deviation of the kurtosis was larger when CV2 was 0.15.

Shojo and Iwaisaki (1999) reported that in a ratio in which the numerator trait constitutes the denominator, the skewness of the ratio trait was positive regardless of the CV of the component traits, but the kurtosis was either positive or negative depending on the CV conditions. In this study, the component traits were assumed to be independent, which may have caused this difference in skewness.

The component traits in this study are assumed to be normally distributed. Because the distribution of the denominator trait (Trait 2) of the component traits is the inverse of a normal distribution, its mean can be regarded as being on a hyperbola. The greater the variance, the wider the left–right range, resulting in a distribution that breaks symmetry and tails to the right. On the other hand, although the numerator trait (Trait 1) is normally distributed, it is affected by the skewness and kurtosis of Trait 2, which influences the skewness and kurtosis of Trait 0. The greater the variance of Trait 1, the more stable the distribution of Trait 0 is expected to be, close to a normal distribution and less affected by Trait 2.

### 3.2 Distribution and Heritability of Ratio Trait With Different Genetic Parameters of Contribution Traits

**FIGURE 2:**
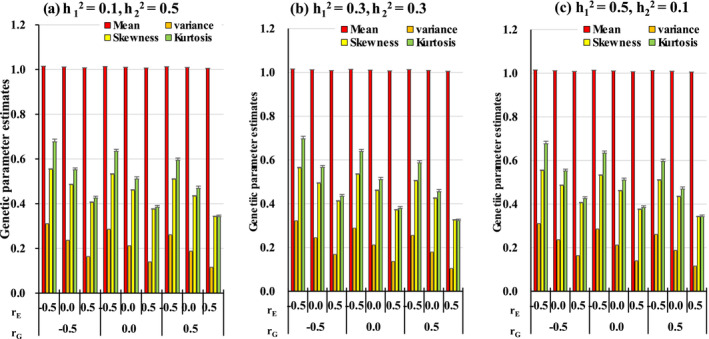
The distribution (mean, variance, skewness, and kurtosis, as well as their standard deviations) of the phenotypic values of the ratio trait (Trait 0) in G1.

Figure 2 shows the distribution (mean, variance, skewness, and kurtosis, as well as their standard deviations) of the phenotypic values of Trait 0 in G1. In this figure, the magnitude of the variance is multiplied by 10. As in Section 3.1, the mean of Trait 0 was similar to the ratio of the means of Traits 1 and 2. The magnitude of the heritability ($h_{1}^{2}$, $h_{2}^{2}$) in Traits 1 and 2 had little effect on the distribution of the phenotypic values of Trait 0, and all of them had the same shape. However, strictly speaking, for example, the means of the variance for $r_{G}=r_{E}=-0.5$ were (a) 0.3103, (b) 0.3221, and (c) 0.3103. Because the standard deviations of all of them were 0.0004, there were statistical differences between (b) and (a) or (c).

As described in Section 2.1, the mean and variance of the ratio trait can be considered as follows. Using the delta method to obtain the expected value and variance of the ratio trait,

(1) $$E\left(p_{0}\right)=E\left(\frac{p_{1}}{p_{2}}\right)≅\frac{\mu_{1}}{\mu_{2}}-\frac{1}{\mu_{2}^{2}}\mathrm{cov}\left(p_{1},p_{2}\right)+\frac{\mu_{1}}{\mu_{2}^{3}}\mathrm{var}\left(p_{2}\right)$$

and

(2) $$\mathrm{var}\left(p_{0}\right)=\mathrm{var}\left(\frac{p_{1}}{p_{2}}\right)≅\frac{1}{\mu_{2}^{2}}\mathrm{var}\left(p_{1}\right)+\frac{\mu_{1}^{2}}{\mu_{2}^{4}}\mathrm{var}\left(p_{2}\right)-\frac{2\mu_{1}}{\mu_{2}^{3}}\mathrm{cov}\left(p_{1},p_{2}\right)$$

are obtained (Appendix A). Briefly, the expectation and variance of the ratio trait can be approximated by the expectation, variance, and covariance of the component traits. Because this study assumes that $\mu_{1}=\mu_{2}=10$, $\sigma_{p_{1}}^{2}=\sigma_{p_{2}}^{2}=1$, from Equations (1) and (2),

$$E\left(p_{0}\right)≅\frac{1}{100}\left[101-\mathrm{cov}\left(p_{1},p_{2}\right)\right]$$

and

$$\mathrm{var}\left(p_{0}\right)≅\frac{2}{100}\left[1-\mathrm{cov}\left(p_{1},p_{2}\right)\right].$$

From $\mathrm{cov}\left(p_{1},p_{2}\right)=r_{g}h_{1}h_{2}+r_{e}\sqrt{\left(1-h_{1}^{2}\right)\left(1-h_{2}^{2}\right)}$, within the setting of this study,

$$-0.5\leq \mathrm{cov}\left(p_{1},p_{2}\right)\leq 0.5,$$

that is,

$$1.005\leq E\left(p_{0}\right)\leq 1.015$$

$$0.01\leq \mathrm{var}\left(p_{0}\right)\leq 0.03.$$

Figure 2 shows that the means and variances of the ratio trait are approximately within this range. Furthermore, their magnitudes are affected only by $r_{g}h_{1}h_{2}+r_{e}\sqrt{\left(1-h_{1}^{2}\right)\left(1-h_{2}^{2}\right)}$. Therefore, even if $r_{G}$ and $r_{E}$ are constant but $h_{1}^{2}$ and $h_{2}^{2}$ are various, the variance of Trait 0 will be affected by $h_{1}^{2}$ and $h_{2}^{2}$. For example, when $r_{G}=r_{E}=-0.5$, the covariance between the component traits is −0.447 for (a) and (c) and −0.500 for (b). Although this difference is very small and cannot be seen clearly on the graph, it is unquestionably statistically significant.

As $r_{G}$ and $r_{E}$ increased, the variance, skewness, and kurtosis of Trait 0 decreased, and the degree of decrease had a greater effect on $r_{E}$ than on $r_{G}$. From Equation (2), the greater the covariance between Traits 1 and 2, the smaller the variance of Trait 0. In the range of heritability used in this study, the genetic covariance between Traits 1 and 2 is the same or smaller than the environmental covariance, so the magnitude of the variance of Trait 0 is more strongly influenced by $r_{E}$ than by $r_{G}$.

Table 1 shows the estimated heritability (${\hat{h}}_{0}^{2}$) and its standard deviation for Trait 0 with various values of $h_{1}^{2}$, $h_{2}^{2}$, $r_{G}$, and $r_{E}$. The value of ${\hat{h}}_{0}^{2}$ decreased as $r_{G}$ increased and decreased as $r_{E}$ decreased. This reason may be explained as follows. From Appendix A,

$$h_{0}^{2}=\frac{\mathrm{var}\left(g_{0}\right)}{\mathrm{var}\left(p_{0}\right)}≅\frac{\mathrm{var}\left(\frac{g_{1}}{g_{2}}\right)}{\mathrm{var}\left(\frac{p_{1}}{p_{2}}\right)}≅\frac{\frac{1}{{\left[E\left(g_{2}\right)\right]}^{2}}\mathrm{var}\left(g_{1}\right)+\frac{{\left[E\left(g_{1}\right)\right]}^{2}}{{\left[E\left(g_{2}\right)\right]}^{4}}\mathrm{var}\left(g_{2}\right)-\frac{2E\left(g_{1}\right)}{{\left[E\left(g_{2}\right)\right]}^{3}}\mathrm{cov}\left(g_{1},g_{2}\right)}{\frac{1}{{\left[E\left(p_{2}\right)\right]}^{2}}\mathrm{var}\left(p_{1}\right)+\frac{{\left[E\left(p_{1}\right)\right]}^{2}}{{\left[E\left(p_{2}\right)\right]}^{4}}\mathrm{var}\left(p_{2}\right)-\frac{2E\left(p_{1}\right)}{{\left[E\left(p_{2}\right)\right]}^{3}}\left[\mathrm{cov}\left(g_{1},g_{2}\right)+\mathrm{cov}\left(e_{1},e_{2}\right)\right]}.$$

**TABLE 1:** Heritability estimate and its standard deviation (× 1000) of ratio trait with various genetic parameters of component traits.

r_G_		−0.5						0.0						0.5					
r_E_		−0.5		0.0		0.5		−0.5		0.0		0.5		−0.5		0.0		0.5	
h_1_^2^ = 0.1	h_2_^2^ = 0.1	0.098	(0.111)	0.141	(0.111)	0.247	(0.131)	0.068	(0.111)	0.098	(0.111)	0.180	(0.121)	0.035	(0.111)	0.052	(0.111)	0.099	(0.111)
	0.3	0.192	(0.131)	0.262	(0.141)	0.413	(0.131)	0.143	(0.131)	0.199	(0.121)	0.330	(0.111)	0.087	(0.121)	0.125	(0.111)	0.219	(0.121)
	0.5	0.285	(0.121)	0.370	(0.121)	0.529	(0.121)	0.226	(0.121)	0.301	(0.131)	0.451	(0.131)	0.156	(0.111)	0.214	(0.111)	0.342	(0.121)
0.3	0.1	0.188	(0.121)	0.257	(0.131)	0.408	(0.121)	0.139	(0.121)	0.195	(0.121)	0.325	(0.131)	0.083	(0.101)	0.120	(0.111)	0.215	(0.131)
	0.3	0.295	(0.121)	0.386	(0.131)	0.555	(0.131)	0.219	(0.121)	0.296	(0.141)	0.456	(0.121)	0.123	(0.121)	0.174	(0.121)	0.296	(0.121)
	0.5	0.395	(0.131)	0.493	(0.121)	0.656	(0.111)	0.307	(0.131)	0.397	(0.131)	0.564	(0.121)	0.187	(0.111)	0.255	(0.111)	0.402	(0.131)
0.5	0.1	0.275	(0.131)	0.360	(0.111)	0.519	(0.121)	0.217	(0.121)	0.291	(0.121)	0.441	(0.131)	0.147	(0.111)	0.204	(0.121)	0.332	(0.121)
	0.3	0.390	(0.121)	0.488	(0.121)	0.651	(0.101)	0.302	(0.131)	0.392	(0.131)	0.559	(0.121)	0.182	(0.121)	0.250	(0.131)	0.398	(0.131)
	0.5	0.493	(0.131)	0.592	(0.131)	0.741	(0.111)	0.394	(0.131)	0.494	(0.131)	0.660	(0.111)	0.247	(0.131)	0.330	(0.121)	0.495	(0.121)

Because this study assumes that $\mu_{1}=\mu_{2}=10$, $\sigma_{p_{1}}^{2}=\sigma_{p_{2}}^{2}=1$,

$$h_{0}^{2}≅\frac{1}{{\left[E\left(g_{2}\right)\right]}^{2}}\cdot \frac{h_{1}^{2}+\frac{{\left[E\left(g_{1}\right)\right]}^{2}}{{\left[E\left(g_{2}\right)\right]}^{2}}h_{2}^{2}-2\frac{E\left(g_{1}\right)}{E\left(g_{2}\right)}h_{1}h_{2}r_{G}}{0.02\left(1-h_{1}h_{2}r_{G}-\sqrt{\left(1-h_{1}^{2}\right)\left(1-h_{2}^{2}\right)}r_{E}\right)}.$$

In addition, the expected breeding value does not need to be zero because the magnitude of breeding value is a relative value. To simplify the discussion, let us assume that $0.01<E\left(g_{1}\right)/E\left(g_{2}\right)$. Therefore, if $r_{G}$ increases, the decrease in the numerator is greater than that in the denominator. As a result, $h_{0}^{2}$ decreases. On the other hand, if $r_{E}$ increases, the denominator decreases. Consequently, $h_{0}^{2}$ is expected to increase.

**FIGURE 3:**
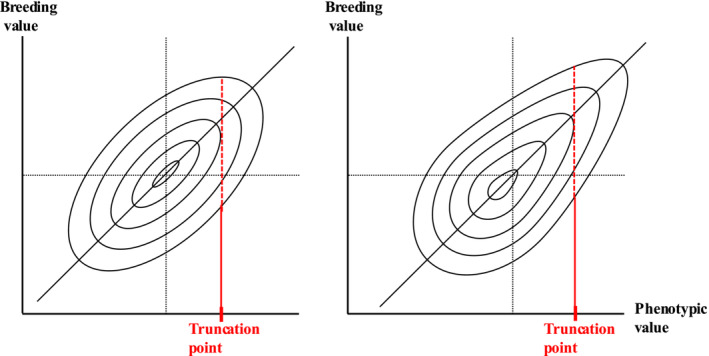
The breeding values and phenotypic values are jointly normally distributed (left) and the joint distribution is skewed (right).

The phenotypic variance of Trait 0 can be obtained from Equation (2). However, although it is possible to estimate the genetic variance and breeding values of Trait 0, it is impossible to obtain their true values even by simulation. Many papers have discussed selection efficiency using component traits and direct selection by the ratio trait (e.g., Famula 1990; Lin and Aggrey 2013; Cerón‐Rojas and Crossa 2020). Iwaisaki and Wilton (1993) theoretically derived a nonlinear regression of the phenotypic value of a ratio trait on the breeding value. In general, index selection by component traits is considered to be more efficient than selection by ratio traits (e.g., Lin and Aggrey 2013; Yazaki et al. 2021a, 2021b). In fact, because selection that imposes restrictions on the genetic gains of traits (Kempthorne and Nordskog 1959; Yamada, Yokouchi, and Nishida 1975; Harville 1975; Tallis 1985) requires the assumption of a multivariate normal distribution for the selected traits, linear selection index and restricted BLUP methods for component traits are effective. However, in selection to maximize aggregate breeding value (Hazel 1943), the normal distribution of the selected traits may not maximize the genetic gain. For example, it is difficult to theoretically clarify the genetic gain due to the same selection rate when the breeding values and phenotypic values are jointly normally distributed and when the joint distribution is skewed (Figure 3). In addition, as shown in Table 1, ${\hat{h}}_{0}^{2}$ can be higher than the heritability of both Traits 1 and 2. In this case, there are no clear criteria for determining the selected traits and their weighting values to maximize the response to selection.

In this study, the author clarified the relationship between the mean and variance of component traits and the distribution of a ratio trait. The author also estimated the heritability of the ratio trait and clarified the relationship between ${\hat{h}}_{0}^{2}$ and the genetic parameters of component traits. Because the distribution and genetic parameters of a ratio trait depend on the genetic parameters of its component traits, it may not be possible to consider all combinations in general terms. It is therefore desirable to target a ratio trait to be genetically improved, such as the feed conversion rate or milk fat percentage, and discuss an optimal improvement method for each trait by investigating the component traits and the relationship between the ratio trait and the component traits.

## Conflicts of Interest

The author declares no conflicts of interest.

## Appendix A

For the two random variables $x=\left(x_{1},\;x_{2}\right)$, the Taylor expansion of the function $y=g\left(x\right)$ of x around the mean $\mu =\left(\mu_{1},\;\mu_{2}\right)$ to the second order term is

$$g\left(x\right)≅g\left(\mu \right)+g_{1}^{\prime }\left(\mu \right)\left(x_{1}-\mu_{1}\right)+g_{2}^{\prime }\left(\mu \right)\left(x_{2}-\mu_{2}\right)+\frac{1}{2}\left[g_{11}^{\prime \prime }\left(\mu \right){\left(x_{1}-\mu_{1}\right)}^{2}+2g_{12}^{\prime \prime }\left(\mu \right)\left(x_{1}-\mu_{1}\right)\left(x_{2}-\mu_{2}\right)+g_{22}^{\prime \prime }\left(\mu \right){\left(x_{2}-\mu_{2}\right)}^{2}\right]$$

where g(x)′ represents the derivative of g(x) with respect to x. Taking the expected values of both sides,

$$E\left[g\left(x\right)\right]≅g\left(\mu \right)+\frac{1}{2}g_{11}^{\prime \prime }\left(\mu \right)\mathrm{var}\left(x_{1}\right)+g_{12}^{\prime \prime }\left(\mu \right)\mathrm{cov}\left(x_{1},x_{2}\right)+\frac{1}{2}g_{22}^{\prime \prime }\left(\mu \right)\mathrm{var}\left(x_{2}\right).$$

The variance of $g\left(x\right)$ $\left[\mathrm{var}\left[g\left(x\right)\right]\right]$ is given by Taylor expansion up to the first order:

$$\mathrm{var}\left[g\left(x\right)\right]≅\mathrm{var}\left[g\left(\mu \right)+g_{1}^{\prime }\left(\mu \right)\left(x_{1}-\mu_{1}\right)+g_{2}^{\prime }\left(\mu \right)\left(x_{2}-\mu_{2}\right)\right]={\left[g_{1}^{\prime }\left(\mu \right)\right]}^{2}\mathrm{var}\left(x_{1}\right)+{\left[g_{2}^{\prime }\left(\mu \right)\right]}^{2}\mathrm{var}\left(x_{2}\right)+2g_{1}^{\prime }\left(\mu \right)g_{2}^{\prime }\left(\mu \right)\mathrm{cov}\left(x_{1},x_{2}\right).$$

If

$$g\left(x\right)=\frac{x_{1}}{x_{2}},$$

$$g_{1}^{\prime }\left(\mu \right)=\frac{1}{\mu_{2}},g_{2}^{\prime }\left(\mu \right)=-\frac{\mu_{1}}{\mu_{2}^{2}},g_{11}^{\prime \prime }\left(\mu \right)=0,g_{12}^{\prime \prime }\left(\mu \right)=-\frac{1}{\mu_{2}^{2}},g_{22}^{\prime \prime }\left(\mu \right)=2\frac{\mu_{1}}{\mu_{2}^{3}}.$$

Therefore, the expected value of $g\left(x\right)$ is

$$E\left[\frac{x_{1}}{x_{2}}\right]≅\frac{\mu_{1}}{\mu_{2}}-\frac{1}{\mu_{2}^{2}}\mathrm{cov}\left(x_{1},x_{2}\right)+\frac{\mu_{1}}{\mu_{2}^{3}}\mathrm{var}\left(x_{2}\right).$$

Also,

$$\mathrm{var}\left[\frac{x_{1}}{x_{2}}\right]≅\frac{1}{\mu_{2}^{2}}\mathrm{var}\left(x_{1}\right)+\frac{\mu_{1}^{2}}{\mu_{2}^{4}}\mathrm{var}\left(x_{2}\right)-\frac{2\mu_{1}}{\mu_{2}^{3}}\mathrm{cov}\left(x_{1},x_{2}\right).$$
